# Cathepsin G Induces Cell Aggregation of Human Breast Cancer MCF-7 Cells via a 2-Step Mechanism: Catalytic Site-Independent Binding to the Cell Surface and Enzymatic Activity-Dependent Induction of the Cell Aggregation

**DOI:** 10.1155/2012/456462

**Published:** 2012-07-08

**Authors:** Riyo Morimoto-Kamata, Sei-ichiro Mizoguchi, Takeo Ichisugi, Satoru Yui

**Affiliations:** Laboratory of Host Defense, Department of Pharma-Sciences, Teikyo University, 2-11-1 Kaga, Itabashi-ku, Tokyo 173-8605, Japan

## Abstract

Neutrophils often invade various tumor tissues and affect tumor progression and metastasis. Cathepsin G (CG) is a serine protease secreted from activated neutrophils. Previously, we have shown that CG induces the formation of E-cadherin-mediated multicellular spheroids of human breast cancer MCF-7 cells; however, the molecular mechanisms involved in this process are unknown. In this study, we investigated whether CG required its enzymatic activity to induce MCF-7 cell aggregation. The cell aggregation-inducing activity of CG was inhibited by pretreatment of CG with the serine protease inhibitors chymostatin and phenylmethylsulfonyl fluoride. In addition, an enzymatically inactive S195G (chymotrypsinogen numbering) CG did not induce cell aggregation. Furthermore, CG specifically bound to the cell surface of MCF-7 cells via a catalytic site-independent mechanism because the binding was not affected by pretreatment of CG with serine protease inhibitors, and cell surface binding was also detected with S195G CG. Therefore, we propose that the CG-induced aggregation of MCF-7 cells occurs via a 2-step process, in which CG binds to the cell surface, independently of its catalytic site, and then induces cell aggregation, which is dependent on its enzymatic activity.

## 1. Introduction 

 Cathepsin G (CG) is a serine protease that is secreted from activated neutrophils and a subset of monocytes, and belongs to the chymotrypsin superfamily [1–4]. Human CG is synthesized as a 255-amino acid-long prepropeptide that contains a signal peptide (Met1-Ala18) followed by a dipeptide (Gly19, Glu20) both of which are removed from the prepropeptide in the endoplasmic reticulum [5]. The mature CG is stored in azurophil granules before degranulation. CG plays important roles not only in the hydrolysis of the extracellular matrix and microbicidal system but also in immune response, apoptosis, chemotaxis, and blood coagulation [1, 3–7]. During infection, CG and other serine proteases, such as neutrophil elastase and proteinase 3, act in conjunction with reactive oxygen species to help degrade engulfed microorganisms inside phagolysosomes [1, 3, 8]. In human leukemic NB4 cells, CG cleaves the protein highly homologous to the *Drosophila* protein “brahma” (brm), which regulates chromatin conformation and the nuclear matrix during apoptosis [9]. In rodent cardiomyocytes, CG promotes detachment-induced apoptosis via a protease-activated-receptor- (PAR-) independent mechanism [10]. In addition, CG is reported to facilitate and impede blood coagulation [6], and it can therefore be considered a regulatory factor in inflammatory and apoptotic reactions. 

 Dissemination of tumor cells from a tumor mass is the first essential step in metastasis [11–13]. The typical disseminating process in tumor metastasis occurs after multiple mutations and the acquisition of highly metastatic properties. These properties include lost capacity for homotypic adherence, gain of high motility, and expression of proteases such as matrix metalloproteases (MMPs), which enable the tumor cells to infiltrate blood vessels and surrounding tissues [12]. Clinical and experimental observations suggest that tumor cells lose their capacity for adherence to the extracellular matrix and form multicellular aggregates, which results in the dissemination of tumor cells from the tumor mass [11, 14]. Subsequently, the multicellular aggregates or spheroids escape from the primary tissues and form emboli in blood vessels or lymph nodes [15–17]. Therefore, it has been speculated that homotypic aggregation is also an important element in the first step of metastasis. However, the physiological factors that modulate the adherence capacity of tumor cells in a tumor environment are poorly understood. 

 Given that leukocytes, including neutrophils, infiltrate and accumulate in tumor masses [18–21], it is important to investigate leukocyte products that regulate the adherence capacity of tumor cells [22]. We previously identified CG as a molecule that induces mammary tumor MCF-7 cells to exhibit tight E-cadherin-mediated cell-cell adhesion following multicellular spheroid formation [23, 24]. We propose that signal transduction events are involved in the reaction, because the guanylate cyclase inhibitor LY83583 had an inhibitory effect on CG-induced MCF-7 aggregation [24]. Moreover, further research is required to elucidate the molecular mechanisms involved in the induction and subsequent aggregation of tumor cells.

 In this study, we show that CG binds to the cell surface of MCF-7 cells and that the MCF-7 cell aggregation-inducing activity of CG requires its enzymatic activity. Interestingly, our analyses of the purified CG protein from neutrophils indicate that the binding of CG to the MCF-7 cell surface is independent of its catalytic site. These results suggest that CG secreted from invading neutrophils may help cancer cells to metastasize via a 2-step mechanism.

## 2. Materials and Methods

### 2.1. Reagents

 CG purified from human neutrophils (95% purity) was purchased from BioCentrum (Kraków, Poland). Anti-CG goat polyclonal antibody and horseradish-peroxidase- (HRP-) conjugated secondary antibodies were obtained from Santa Cruz Biotechnology (Santa Cruz, CA, USA). Anti-*β*-actin mouse monoclonal antibody, bovine pancreatic chymotrypsin, *α*_1_-antitrypsin (AT), and phenylmethylsulfonyl fluoride (PMSF) were from Sigma (St. Louis, Mo, USA). Chymostatin and *α*_1_-antichymotrypsin (ACT) were purchased from Calbiochem (Darmstadt, Germany). Suc-Val-Pro-Phe^P^-(OPhe)_2_ was kindly donated by Dr. Jozef Oleksyszyn (University Wroclam, Poland). PAR ligands (PAR-1 ligand, H-Ser-Phe-Leu-Arg-Asn-NH_2_; PAR-2 ligand, H-Ser-Leu-Ile-Gly-Lys-Val-NH_2_; PAR-4, H-Ala-Tyr-Pro-Gly-Lys-Phe-NH_2_) were purchased from Sigma. PMSF-treated CG was prepared by incubating CG (final concentration, 8.34 *μ*M) with PMSF (4 mM) for 2 h at 37°C followed by dialysis to remove unbound PMSF that would cause cytotoxicity in MCF-7 cells in cell aggregation assays.

### 2.2. Cell Culture

 Human breast cancer MCF-7 cells were kindly provided by Dr. Hiroshi Kosano (Teikyo University, Japan). The MCF-7 cells were maintained in RPMI 1640 medium supplemented with 10% heat-inactivated fetal bovine serum (FBS; MP Biomedicals, Solon, OH, USA) and 80 *μ*g/mL kanamycin (Wako Pure Chemical, Osaka, Japan) as described previously [24]. The rat basophilic/mast cell line RBL-2H3 was purchased from the Riken Cell Bank (Tsukuba, Japan), and the cells were maintained in Eagle's minimum essential medium supplemented with 10% FBS and 80 *μ*g/mL kanamycin. The cells were incubated at 37°C in a humidified atmosphere of 5% CO_2_. 

### 2.3. MCF-7 Cell Aggregation Assay

 To quantitatively assess the degree of spheroid formation, we quantified the cells that were tightly attached to the culture plate by staining with crystal violet as previously described [23]. MCF-7 cells (1 × 10^4^ cells/well) were seeded in 96-multiwell plates and cultured for 24 h. The cells were seeded in RPMI 1640 medium containing 5% FBS and then washed with serum-free RPMI 1640 medium. The medium was then replaced with RPMI 1640 medium containing 1% bovine serum albumin (BSA) and diluted purified CG or lysates of RBL-2H3 cells overexpressing *CG*. After cultivation for 24 h, the plate was vigorously tapped on paper towels 10 times to eliminate the loosely attached cell spheroids. The remaining cells were then dried at room temperature and stained with 0.1% crystal violet in phosphate-buffered saline (PBS) for 10 min. Following this, the plate was extensively washed with tap water. The plate was then dried at room temperature, and the crystal violet in the residual cells was lysed with 100 *μ*L of 0.5% sodium dodecyl sulfate (SDS). Optical density at 595 nm (OD_595_) was measured with a microplate reader (Multiskan MS-UV; Labsystems, Thermo Fisher Scientific, Waltham, MA, USA), and the aggregation index was calculated as follows:

(1) aggregation  index  (%) =OD595  without  sample−OD595  with  test  samplesOD595  without  sample  ×100.

Although some calculated values for the aggregation index were slightly below zero, microscopic observation revealed that the cells were morphologically similar to nonaggregated control cells, and therefore the negative values are expressed as zero in the figures to avoid confusion.

### 2.4. Measurement of CG Enzymatic Activity

 The enzymatic activity of CG was measured using an established method [25], with *N*-succinyl-Ala-Ala-Pro-Phe *p*-nitroanilide (Sigma) as a substrate. One unit represents the amount of enzyme that hydrolyzes 1.0 *μ*mol of the substrate per minute at 25°C at pH 7.5.

### 2.5. Construction of Expression Vectors

 The pEGFP-N3 and pcDNA3.1-Hyg (−) plasmids were purchased from Life Technologies Corporation (Grand Island, NY, USA). Human *CG* cDNA (Genbank Acc. BC014460) encoded in pENTR221 was purchased from Promega (Madison, WI, USA). The *CG* cDNA was amplified by PCR, and the cDNA fragment containing the open reading frame region of the *CG* gene was subcloned into the *Kpn*I and *Eco*RI sites of pcDNA3.1. The primer sequences were 5′-AAAAAGGTACCATGCAGCCACTCCTGCTTC-3′ and 5′-AAAAAGAATTCTACAGGGGGGTCTCCATCT-3′ (start and stop codons are underlined). The CG S195G (chymotrypsinogen numbering, amino acid residue S201 of pre-proCG) mutant was generated using the PrimeSTAR mutagenesis basal kit (TaKaRa Bio, Shiga, Japan). The primer sequences used for mutagenesis were 5′-GGGGATGGCGGAGGCCCCCTGCTGTGT-3′ and 5′-GCCTCCGCCATCCCCCTTGAAGGCAGCCTTCCG-3′ (mutated bases are underlined). Sequences of the wild-type (WT) and S195G mutant *CG* cDNAs were confirmed by sequencing using an ABI3130 genetic analyzer (Life Technologies Corporation). 

### 2.6. Transfection

 Transient overexpression of the *CG* gene in RBL-2H3 cells was achieved by electroporation. Briefly, the cells were harvested by treatment with PBS containing 0.53 mM EDTA and 0.25% trypsin (BD Difco, Franklin Lakes, NJ, USA). After digestion, the cells were washed once with PBS and twice with Opti-MEM (Life Technologies Corporation). The cells (1 × 10^6^ cells) and plasmid (10 *μ*g) were mixed and pulsed at 275 V for 3 ms using the CUY21 Pro-Vitro electroporation system (NEPAGENE, Chiba, Japan). These cells were used in the following experimental procedures at 24 h after transfection.

### 2.7. Western Blotting

 SDS-polyacrylamide gel electrophoresis (SDS-PAGE) and subsequent western blotting were performed as previously described [23]. The cell lysates were prepared by sonication in PBS after the addition of an equal volume of 2x sample buffer (100 mM Tris-HCl, pH 6.8 containing 2% 2-mercaptoethanol, 2% SDS, 40% glycerol, and 0.02% Coomassie Brilliant Blue) and boiled for 5 min. The samples were separated by SDS-PAGE using a precast 15% Tris-tricine gel (Atto Corporation, Tokyo, Japan) and were transferred onto polyvinyl difluoride membranes (GE Healthcare, Buckinghamshire, UK). After blocking by incubation with Tris-buffered saline (TBS) containing 0.1% Tween 20 (TBS-T) and 5% ECL blocking agent (GE Healthcare) for 1 h, the membrane was reacted with an anti-CG goat polyclonal antibody at 4°C and extensively washed with TBS-T. The membrane was then incubated with HRP-labeled secondary antibody (Santa Cruz), washed, developed by incubation with ECL detection reagents (GE Healthcare), and exposed to Hyperfilm ECL (GE Healthcare). CG expression levels were quantified using ImageQuant TL (GE Healthcare). 

### 2.8. Immunohistochemistry

 Cells were fixed with 4% paraformaldehyde in PBS for 30 min followed by blocking in PBS containing 2% BSA (blocking buffer) for 1 h. The cells were reacted with anti-CG antibody (1 : 10) in blocking buffer overnight at 4°C without permeabilization. The samples were washed 4 times with PBS and reacted with anti-goat secondary antibodies conjugated to Alexa Fluor 568 (1 *μ*g/mL; Life Technologies Corporation). The samples were then washed 4 times with PBS, and the immunoreactivity was observed under a fluorescent microscope (Olympus Corporation, Tokyo, Japan). 

### 2.9. Biotinylation and Purification of Cell Surface Proteins

 Biotinylation of cell surface proteins was performed as previously described [26]. MCF-7 cells (1 × 10^4^ cells/dish) were treated with lysates of human CG-expressing RBL-2H3 cells on ice for 90 min and then biotinylated with 0.5 mg/mL EZ-Link NHS-biotin (Thermo Scientific Pierce, Waltham, MA, USA) in PBS at 4°C for 1 h. The reaction was stopped by the addition of 25 mM L-lysine, and the harvested cells were extracted in a buffer containing 25 mM Tris-HCl (pH 8.0), 1% Triton X-100, and 100 mM NaCl. The extracts were incubated with avidin-agarose beads (Sigma), and the precipitated proteins were immunoblotted with anti-CG antibody. 

### 2.10. Covalent Complex Formation between CG and AT or ACT

 CG purified from neutrophils (1.67 pmol) was incubated with AT (90.9 pmol) or ACT (1.5 pmol) in RPMI 1640 medium containing 1% BSA for 10 min at 4°C. In addition, to measure the effect of an inhibitor on complex formation, CG was pretreated with chymostatin (661 pmol). Formation of the covalent complex in the mixture was determined by the presence of a heavier CG band on the western blot membrane. 

### 2.11. Binding Assay Using ^125^I-Labeled Serine Proteases

 Serine proteases were radiolabeled by chloramine T-mediated ^125^I-iodination [27]. CG was dissolved in 0.1 M sodium phosphate buffer containing Na ^125^I (Perkin-Elmer, Waltham, MA, USA) and was oxidized by the addition of 1 mg/mL chloramine T. Subsequently, the residual unreacted chloramine T was reduced with sodium pyrosulfite. For stabilization, 50 mM KI and 0.5% BSA were added to the ^125^I-labeled CG solution. ^125^I-CG was separated from the residual unreacted ^125^I and concentrated using a D-Salt Excellulose desalting column (Pierce). The specific activity of ^125^I-labeled CG was measured using a *γ*-counter (Aloka Auto well gamma system ARC-300; Hitachi Aloka Medicals, Ltd., Tokyo, Japan) and was approximately 1.25 × 10^6^ Bq/*μ*g. To measure the binding of the serine proteases to the MCF-7 cell surface, round-bottomed 96-well plates containing RPMI 1640 medium with 5% FBS were seeded with MCF-7 cells (2 × 10^4^ cells/well). After washing with RPMI 1640 medium containing 1% BSA, the cells were incubated with ^125^I-labeled serine proteases for 1 h on ice. Unbound serine proteases were removed by washing the cells thrice with RPMI 1640 medium containing 1% BSA and thrice with PBS. The radioactivity of the cell lysate, which was prepared using 0.1 M NaOH, was measured using the *γ*-counter. In the time course experiment, 20-*μ*L aliquots of ^125^I-labeled CG were added to the MCF-7 cells. The protein concentration was determined using the bicinchoninic acid (BCA) protein assay kit (Pierce) with BSA as a standard. 

### 2.12. Statistical Analysis

 For statistical analysis of the data, Student's *t*-tests were used. Data are expressed as mean (standard deviation (SD)), unless indicated otherwise. The data of the enzymatic activity assays in Figure 2 and the data of Figure 6(a) are single-point values.

## 3. Results

### 3.1. Enzymatic Activity of CG Is Required for Its MCF-7 Cell Aggregation-Inducing Activity 

**Figure 1:**
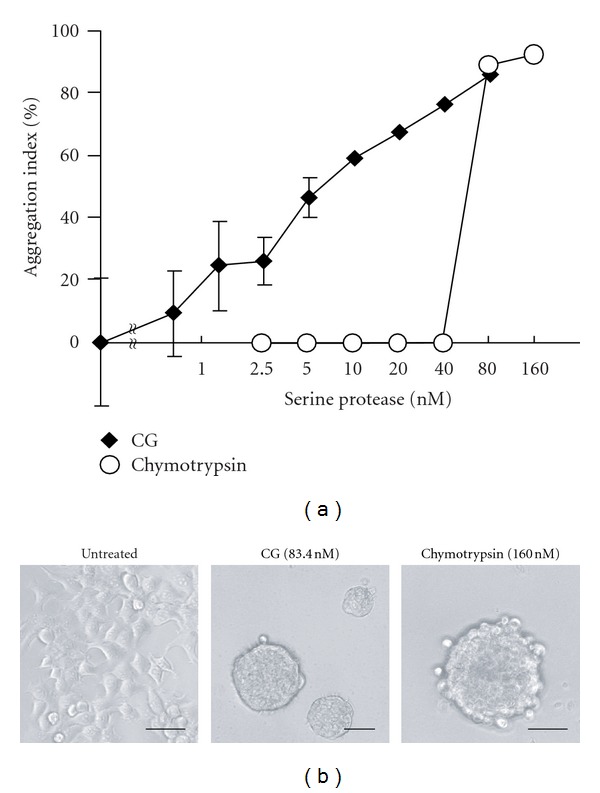
MCF-7 cell aggregation-inducing activities of cathepsin G (CG) and chymotrypsin. (a) MCF-7 cell aggregation assay using CG and chymotrypsin. MCF-7 cells (1 × 10^4^ cells/well) were seeded in 96-well plates in RPMI 1640 medium containing 5% fatal bovine serum (FBS). The cells were cultured overnight and then incubated overnight with the serine proteases in RPMI 1640 medium containing 1% BSA. After washing, the residual cells were stained with crystal violet, and the aggregation index was calculated as described in Section 2. The results are expressed as mean ± SD (*n* = 3). When the bars are not shown, they are smaller than the size of the symbols. (b) Images of MCF-7 cells at 24 h after incubation with the serine proteases. Scale bar = 50 *μ*m.

 We have previously demonstrated that CG induces homotypic cell aggregation and the formation of multicellular 3D spheroids of MCF-7 cells [23, 24]. To elucidate the molecular mechanism by which CG induced MCF-7 cell aggregation, we compared the cell aggregation-inducing activity of CG with that of chymotrypsin, because CG belongs to the chymotrypsin superfamily. Figure 1(a) shows that CG induced MCF-7 cell aggregation in a linear dose-dependent manner, whereas chymotrypsin was effective only at 80 nM. Both CG and chymotrypsin stimulated the cells to condense into similar multicellular spheroids (Figure 1(b)). Chymotrypsin induced less cell aggregation than CG, although chymotrypsin had a higher enzymatic activity (2370 U/mg) than CG (99 U/mg) when *N*-succinyl-Ala-Ala-Pro-Phe *p*-nitroanilide was used as the substrate.

**Figure 2:**
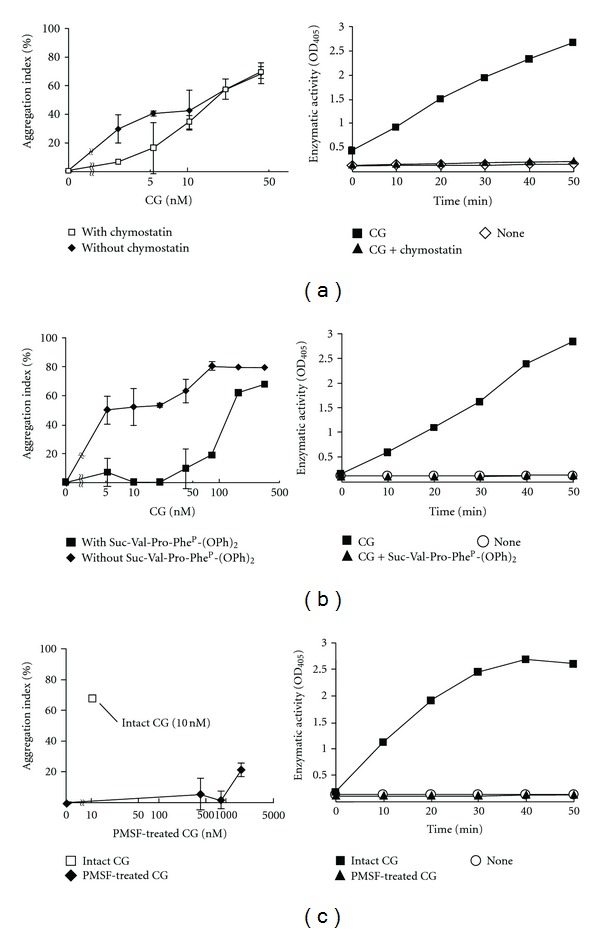
The MCF-7 cell aggregation-inducing activity of CG is inhibited by serine protease inhibitors. CG was simultaneously added to the medium with the serine protease inhibitor chymostatin (16.5 *μ*M) (a) or Suc-Val-Pro-Phe^P^-(OPh)_2_ (10 *μ*M) (b). PMSF-treated CG was added to MCF-7 cells (c). The aggregation index is shown in the left panels of Figures 2(a), 2(b), and 2(c). The results are shown as mean ± SD (*n* = 3). When the bars are not shown, they are smaller than the size of the symbols. The inhibitory effect of the serine protease inhibitors on the enzymatic activity of CG is also shown (right panels). The enzymatic activity of CG was analyzed by measuring the release rate of 4-nitroanilide following the addition of CG (667 nM, right panels of (a) and (b)) and the inhibitors (16.5 *μ*M chymostatin, right panel of (a); 10 *μ*M Suc-Val-Pro-Phe^P^-(OPh)_2_, right panel of (b)) to *N*-succinyl Ala-Ala-Pro-Phe *p*-nitroanilide (1.1 mg/mL) in 0.1 M HEPES buffer (pH 7.5) containing 0.5 M NaCl and 10% dimethyl sulfoxide at 25°C. The released *p*-nitroanilide was detected by measuring the absorbance at 405 nm. In the right panel of (c), the effect of 417 nM intact or PMSF-treated CG was measured. The data of the enzymatic activity are indicated as single-point values.

 Next, we examined whether the enzymatic activity of CG was essential for cell aggregation by treating MCF-7 cells with various serine protease inhibitors. We have previously shown that the cell aggregation is inhibited by treatment with the serine protease inhibitors AT and ACT, which are members of the serine protease inhibitor (serpin) superfamily and are composed of 300–500 amino acid residues [3, 23, 28]. However, because AT and ACT irreversibly disrupt the CG conformation by forming a covalent complex with CG, the inhibition of CG-induced MCF-7 aggregation by AT and ACT results from both enzymatic inactivation and steric hindrance preventing interaction with the target molecule. To separate these effects, we used peptidic serine protease inhibitors. The reversible inhibitor chymostatin (16.5 *μ*M), which is a tetrapeptide analog of the CG substrate, slightly inhibited MCF-7 cell aggregation when incubated with 2.63–5.25 nM CG (Figure 2(a)). Treatment with another serine protease inhibitor Suc-Val-Pro-Phe^P^-(OPh)_2_, which is a moderately irreversible *α*-aminoalkylphosphonate diphenyl ester inhibitor, more potently decreased the cell aggregation (Figure 2(b)). To support these results, we used the low-molecular-weight and irreversible serine protease inhibitor PMSF. To avoid PMSF-induced cytotoxicity, we used PMSF-treated CG, which was prepared by incubating CG with PMSF, followed by dialysis to remove the unbound PMSF. PMSF-treated CG (<167 nM) markedly inhibited the cell aggregation (Figure 2(c)). 

 To clearly and directly demonstrate the importance of enzymatic activity in cell aggregation, we prepared the recombinant S195G (chymotrypsinogen numbering; Ser201 residue of pre-pro CG) CG protein. The S195G CG protein, in which a serine of the catalytic triad is substituted with glycine, was prepared by overexpression in the rat basophilic/mast cell line RBL-2H3 [29]. RBL-2H3 cells can synthesize the mature CG protein because these cells express signal peptidase and dipeptidyl dipeptidase I for processing pre-proCG but not endogenous CG [3, 30]. Western blot analysis using the anti-CG antibody revealed polypeptides of ~26 kDa in cell lysates prepared from cells transfected with the WT CG- and S195G CG-expressing vectors (see Supplementary Figure 1(b) available online at doi:1155/2012/456462). The lysate from RBL-2H3 cells transfected with the empty vector did not contain the CG polypeptide. The estimated molecular weight of the pre-proCG peptide is 29 kDa, and, therefore, the recombinant CG synthesized in the RBL-2H3 cells had probably undergone posttranslational processing. We noted that the CG peptide in the transfected RBL-2H3 cell lysates migrated at a slightly higher position on the SDS-PAGE gel than the CG purified from human neutrophils (see Supplementary Figure 1(b)). The higher shift of the recombinant CG on the SDS-PAGE is because of incomplete cleavage of the C terminus, which does not influence the enzymatic activity [31, 32].

 Subsequently, we analyzed the enzymatic activity of CG in the lysates using *N*-succinyl-Ala-Ala-Pro-Phe *p*-nitroanilide. The enzymatic activity, which was normalized to *β*-actin expression, was 73.4% and 70.1% in the lysates from S195G CG-transfected and control vector-transfected cells, respectively, compared to that in the lysate containing WT CG (see Supplementary Figures 1(a) and 1(c)). The relatively high enzymatic activity of the control vector-transfected cells may be derived from the intact RBL-2H3 cells, because these cells have been reported to express a chymotrypsin-like enzyme that hydrolyzes *N*-succinyl Ala-Ala-Pro-Phe *p*-nitroanilide [33–35]. Furthermore, RBL-2H3 cells express the chymotrypsin-type serine proteases rat mast cell protease (rMcp)-2 and rMcp3 but not chymase [35, 36]. Therefore, we concluded that the recombinant WT CG protein was enzymatically active, whereas S195G CG was inactive. 

**Figure 3:**
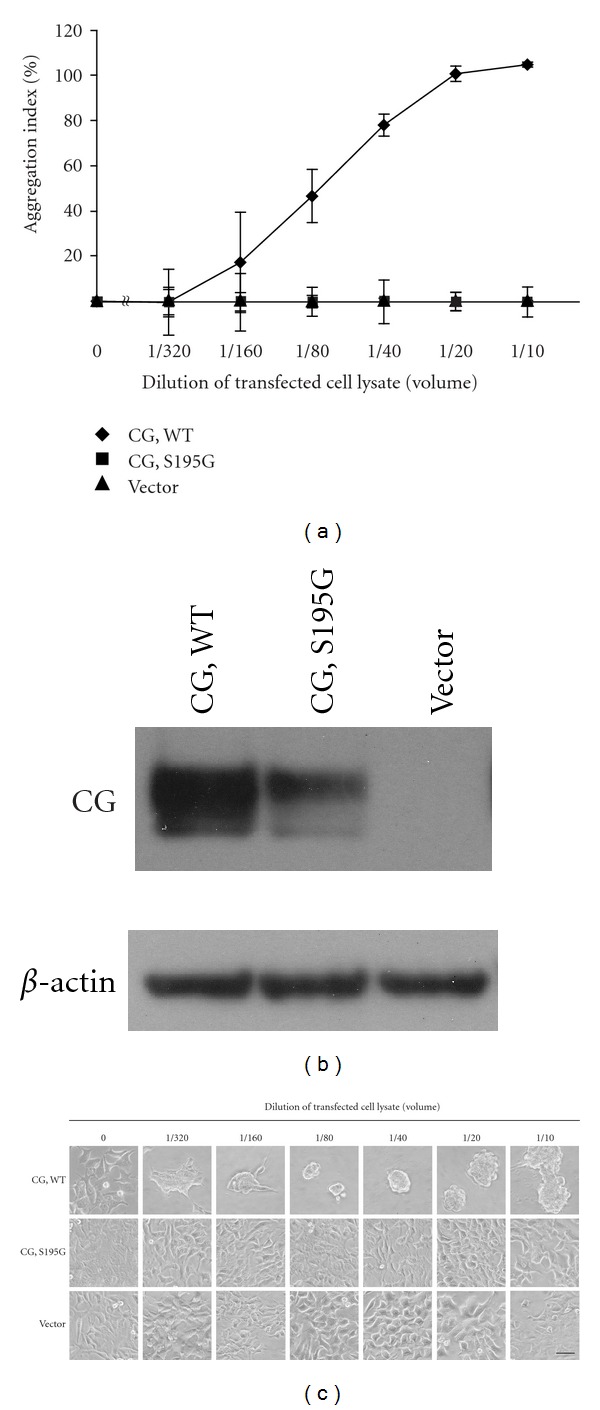
MCF-7 cell aggregation is induced by wild-type (WT) CG, but not the enzymatically inactive S195G mutant. (a) MCF-7 cell aggregation assay using CG-overexpressing cell lysates. MCF-7 cells were cultured overnight in medium containing 5% FBS and incubated with lysates serially diluted in serum-free medium containing 1% BSA. After washing, the residual cells were quantified by crystal violet staining. The results are shown as mean ± SD (*n* = 3). When the bars are not shown, they are smaller than the size of the symbols. (b) Immunoreactivities of CG and *β*-actin in the lysates assayed in Figure 3(a). A 10-*μ*L aliquot of the whole cell lysate was analyzed by western blotting using anti-CG or *β*-actin antibody. (c) Images of cells that were analyzed in Figure 3(a) at 24 h after incubation with the diluted lysates. Scale bar = 50 *μ*m.

 Analyses of cell aggregation using lysates prepared from CG-expressing RBL-2H3 cells and MCF-7 cells revealed that the WT CG-expressing cell lysate induced cell aggregation in a dose-dependent manner (Figure 3). The resultant multicellular 3D-spheroids were morphologically similar to those induced by the CG purified from neutrophils (Figures 1(b) and 3(c)). By contrast, the lysates derived from S195G CG-expressing cells and vector-transfected cells did not induce cell aggregation. These findings indicate that the enzymatic activity of CG is required for CG-induced MCF-7 cell aggregation. 

### 3.2. CG Binds to the Surface of MCF-7 Cells Independently of Its Catalytic Site

**Figure 4:**
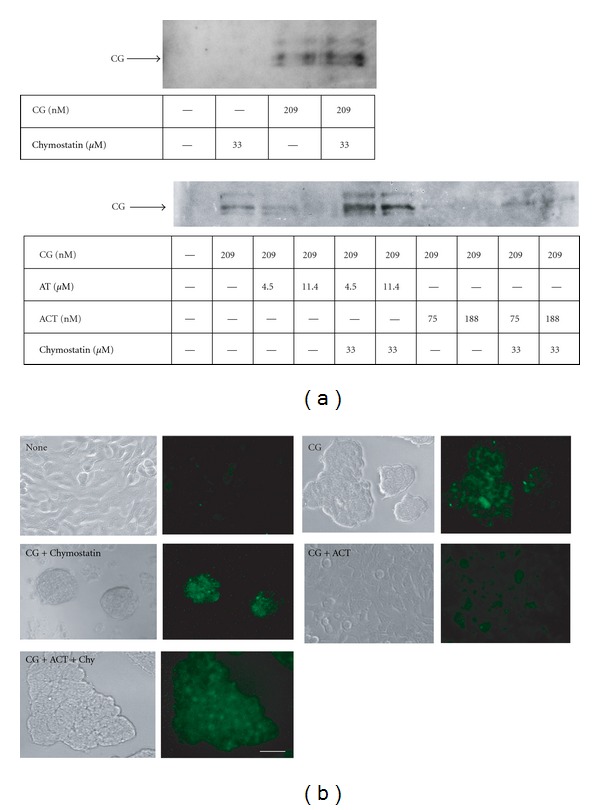
CG binds to the surface of MCF-7 cells. (a) Detection of CG purified from human neutrophils in the MCF-7 cell surface fraction. MCF-7 cells were seeded in dishes containing RPMI 1640 medium supplemented with 5% FBS. The cells were cultured overnight and subsequently incubated with CG (209 nM) and chymostatin (33 *μ*M), AT (4.5 or 11.4 *μ*M), or ACT (75 or 188 nM) in serum-free RPMI 1640 medium for 90 min on ice. After washing, cell surface proteins were biotinylated and collected using avidin-conjugated agarose beads. The cell surface protein fractions were analyzed by western blot analysis using anti-CG antibody. (b) Immunohistochemical detection of CG on MCF-7 cells. MCF-7 cells that were treated with CG (209 nM) and a protease inhibitor (chymostatin, 16.5 *μ*M; ACT, 18.8 nM) were immunostained with anti-CG antibody in the absence of permeabilization. Scale bar = 20 *μ*m.

**Figure 5:**
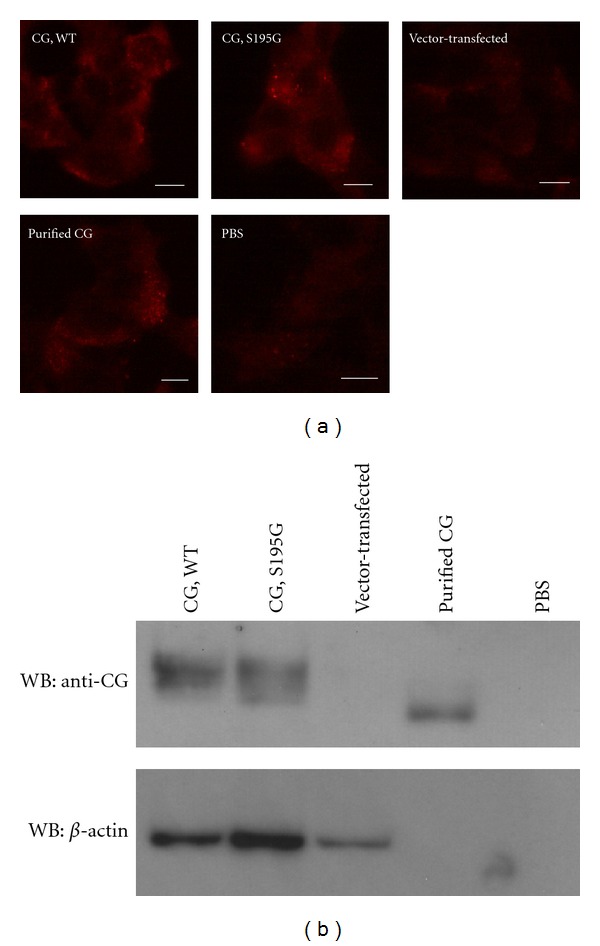
Binding of CG to the surface of MCF-7 cells is independent of its catalytic site. (a) The S195G CG mutant binds to MCF-7 cells. The cells were incubated with CG-overexpressing RBL-2H3 cell lysate or purified human CG from neutrophils (final concentration, 209 nM) for 90 min on ice. The CG bound to the cell surface was detected by immunostaining with anti-CG antibody in the absence of permeabilization. Scale bar = 10 *μ*m. (b) Western blot analysis of CG in the lysates used in Figure 5(a) using anti-CG antibody. A 10-*μ*L aliquot of the lysate was loaded in each lane.

 The target molecule(s) of CG in the induction of cell aggregation remain unclear. If the target molecule of CG localizes to the cell surface of MCF-7 cells, it is possible that CG tethers to the cell surface and proteolytically cleaves the target molecule. Accordingly, we next examined the binding of CG to the cell surface proteins of MCF-7 cells. To this end, biotinylated cell surface proteins prepared from MCF-7 cells pretreated with 209 nM purified CG were precipitated using avidin-conjugated beads. Western blot analysis showed that the precipitate contained the CG protein (Figure 4(a)). To determine whether the binding activity of CG also required its enzymatic activity, chymostatin, AT, or ACT was added to MCF-7 cells along with CG during the binding assay. AT and ACT, but not chymostatin, inhibited the binding of CG to the MCF-7 cell surface (Figure 4(a)). Immunohistochemistry yielded the same result (Figure 4(b)). Interestingly, inhibition of the binding by AT and ACT was blocked by the addition of chymostatin (Figure 4). We propose that this is due to the preferential binding of chymostatin to CG, which inhibits the formation of the AT-CG or ACT-CG complex. Indeed, covalent AT-CG and ACT-CG complexes were detected as 80-kDa polypeptides by western blot analysis using anti-CG antibody, and the formation of these complexes was inhibited by the addition of chymostatin (Supplementary Figure 2). Furthermore, WT and S195G recombinant CG proteins were also detected on nonpermeabilized MCF-7 cells by immunostaining with anti-CG antibody (Figure 5). Thus, these results indicate that CG binds to the surface of MCF-7 cells independently of its catalytic site. 

**Figure 6:**
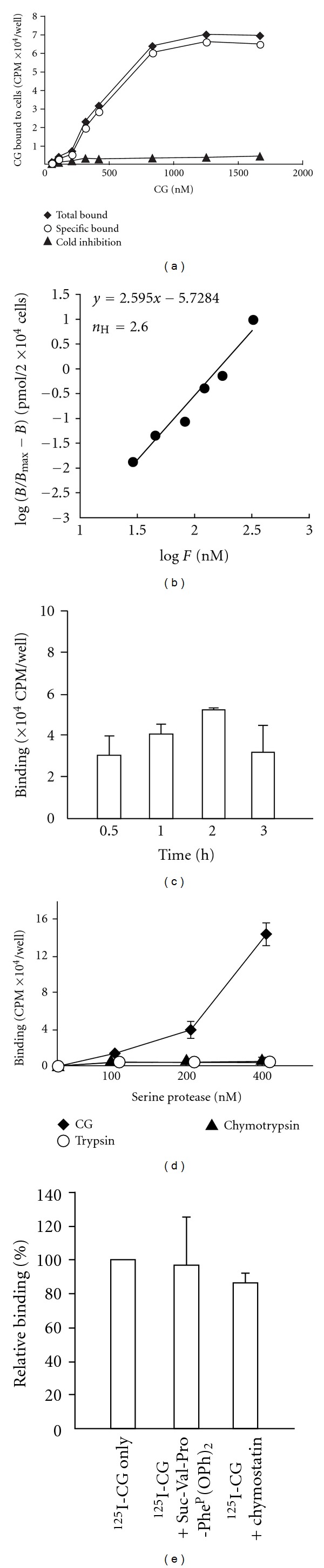
Characterization of the binding of ^125^I-labeled CG to the MCF-7 cell surface. (a) Dose dependency of ^125^I-CG binding to MCF-7 cells. The cells were incubated with ^125^I-labeled CG in RPMI 1640 medium containing 1% BSA for 60 min on ice. After washing, the cells were disrupted by the addition of 0.1 M NaOH, and the radioactivity of the lysate was determined using a *γ*-counter. In the cold inhibition experiment, a 20-fold excess of unlabeled CG was simultaneously added to the medium for competitive binding. Specific binding was determined by subtracting the value obtained for nonspecific binding (cold inhibition) from the total binding. The data are expressed as single-point values. (b) Hill plot analysis of ^125^I-CG binding to MCF-7 cells. The slope of the Hill plot is the Hill coefficient (*n*_H_), which indicates cooperativity. (c) Time course of ^125^I-CG binding to MCF-7 cells. ^125^I-CG was added at a final concentration of 834 nM. (d) Binding activities of ^125^I-trypsin and ^125^I-chymotrypsin. (e) Suc-Val-Pro-Phe^P^-(OPh)_2_ and chymostatin have no effect on CG binding to MCF-7 cells. MCF-7 cells were incubated with ^125^I-CG (83.4 nM) that was pretreated with serine protease inhibitors (Suc-Val-Pro-Phe^P^-(OPh)_2_, 100 *μ*M; chymostatin, 82.5 nM). The bound ^125^I-CG is expressed as relative binding comparing the radioactivity of bound intact ^125^I-CG with that of serine protease-treated ^125^I-CG. Unless otherwise indicated, similar results were obtained from 2 independent experiments, each with duplicates. The results are shown as mean ± SD. When the bars are not shown, they are smaller than the size of the symbols.

 We next analyzed the binding of CG to the MCF-7 cell surface using ^125^I-labeled CG. We confirmed that ^125^I-labeled CG had cell aggregation-inducing activity equivalent to that of intact CG (data not shown). The sigmoidal binding curve and complete inhibition of the binding by the addition of 20-fold excess of cold CG strongly suggested that CG bound to a specific site on the cell surface (Figure 6(a)). Unfortunately, sigmoidal binding curves prevent a precise estimation of the number of binding sites of a ligand using Scatchard plot analysis. Indeed, we were unable to estimate the number of CG-binding sites because the Scatchard plot of CG binding to MCF-7 cells exhibited a convex upward curve (see Supplementary Figure 4). The binding exhibited a positive cooperative effect, as CG binding increased concurrently with increasing CG concentrations. Hill plot analysis enables the assessment of cooperativity between a receptor and ligand, and when the slope of the Hill plot (*n*_H_) is >1, the receptor and ligand exhibit positive cooperativity [27, 29]. The *n*_H_ of our study was 2.6, indicating positive cooperativity (Figure 6(b)). The time course of CG binding demonstrated that the binding reached saturation after 2 h (Figure 6(c)). We also analyzed the binding of ^125^I-chymotrypsin and ^125^I-trypsin to MCF-7 cells. However, these serine proteases were unable to bind to the cells (Figure 6(d)). CG binding was not inhibited by the addition of the peptide inhibitors Suc-Val-Pro-Phe^P^-(OPh)_2_ and chymostatin (Figure 6(e)). These data suggest that CG is specifically bound to a cell surface protein via a catalytic site-independent mechanism. 

### 3.3. CG-Induced MCF-7 Cell Aggregation Is Not Mediated by PAR-1, PAR-2, or PAR-4 

 We sought to identify the target protein of CG on MCF-7 cells. PARs belong to a novel subfamily of G-protein-coupled receptors that are proteolytically activated by the unmasking of an N-terminal tethered ligand sequence by serine proteases [37, 38]. MCF-7 cells express trace amounts of PAR1 and low levels of PAR-2 and PAR-4. In addition, PAR-1 has been shown to inhibit the migration and invasion of MCF-7 cells, whereas PAR-2 and PAR-4 positively regulate these functions [39]. To determine the relationship between PARs and the MCF-7 cell aggregation induced by CG, we stimulated MCF-7 cells with the PAR ligands. Incubation with the PAR-1 or PAR-2 ligand (up to 150 *μ*M) did not induce cell aggregation, and the PAR-4 ligand elicited a slight aggregation of MCF-7 cells in a dose-independent manner (see Supplementary Figure 3). No morphological changes were observed in MCF-7 cells in the presence of the PAR agonists (data not shown). These findings were supported by the observation that MCF-7 cell aggregation was not induced by thrombin, which is known to be a strong ligand for PAR-1, PAR-3, and PAR-4 (data not shown). Accordingly, we concluded that CG binds to the MCF-7 cell surface, independently of its catalytic site and enzymatic activity, and subsequently induces MCF-7 cell aggregation in an enzymatic activity-dependent manner. In addition, the CG-induced cell aggregation is not mediated by PARs.

## 4. Discussion

 Previously, we showed that CG induces the formation of multicellular MCF-7 aggregates, as a result of increased cell motility and switching from cell-extracellular matrix adhesion to E-cadherin-mediated cell-cell adhesion [23, 24]. We also demonstrated that the cell condensation-inducing activity of CG is inhibited by treatment with the serine protease inhibitors AT and ACT; however, this observation is not direct evidence of a relationship between the enzymatic activity and the cell aggregation-inducing activity of CG because AT and ACT are known to form covalent complexes with serine proteases, and the complex may prevent the interaction between CG and its target molecule on MCF-7 cells via steric hindrance. In this study, we used a peptidic small molecule serine protease inhibitor and an enzymatically inactive mutant of the recombinant CG protein to clearly demonstrate that CG requires its enzyme activity for the induction of cell aggregation. 

 Although the substrate specificities of these serine proteases are similar [3, 25, 40], the binding and aggregation-inducing characteristics of CG and chymotrypsin were different. CG induced MCF-7 cell aggregation in a linear dose-dependent manner, whereas chymotrypsin required a concentration >80 nM. CG associated with MCF-7 cell surface proteins, whereas chymotrypsin did not. The specificities of CG and chymotrypsin are slightly different: chymotrypsin recognizes aromatic residues such as Phe, Trp, and Tyr at the P1 position as a substrate, whereas CG prefers aromatic or positively charged residues such as Phe, Tyr, Lys, and Arg. This difference in substrate specificity may be important for the CG-specific induction of cell aggregation. 

 It is important to identify the target molecule(s) and the CG signaling pathways in MCF-7 cells. Our results suggest that the target of CG is a receptor or an adhesion molecule. We had predicted that the PARs may be a candidate for the CG receptor. MCF-7 cells express trace amounts of PAR-1 and low levels of PAR-2 and PAR-4 [39]. CG activates PAR-4 in addition to thrombin to initiate thrombocyte aggregation [1, 41]. In addition, CG recruits osteoclast precursors via the proteolytic activation of PAR-1 [42]. However, incubation with <150 *μ*M ligand peptides that proteolytically cleave PAR-1, PAR-2, and PAR-4 failed to induce cell aggregation; thus, the CG target remains unidentified. In human neutrophils, CG functions as a ligand of the formyl Met-Leu-Phe peptide (fMLP) receptor and induces chemotaxis [43]. However, given that the addition of fMLP did not inhibit the CG-induced cell aggregation, we conclude that the fMLP receptor is not associated with cell aggregation (data not shown). We have unpublished data that CG has no effect either on cell detachment or on spheroid formation of human cervical adenocarcinoma HeLa cells and human fibrosarcoma HT1080 cells (data not shown). CG induces the detachment of E-cadherin-deficient human breast cancer MDA MB-231 cells, but not their aggregation (unpublished observation). ^125^I-CG also binds to MDA MB-231 cells (unpublished observation). These results raise the possibility that the binding target(s) of CG and the signaling molecules are specifically expressed in at least several types of breast cancer cells, and the induction of cell aggregation depends on the presence of E-cadherin molecules. 

 In the present study, we have also demonstrated the binding of CG to the MCF-7 cell surface. Chymotrypsin did not bind to the cells. Membrane association of CG has been reported in human neutrophils [44]. The mechanism through which CG is associated with the outer surface of the plasma membrane of neutrophils mainly involves electrostatic interactions with the sulfate groups of chondroitin sulfate- and heparin sulfate-containing proteoglycans [45]. CG contains 17.3% basic amino acid residues and, therefore, is a highly cationic protein. Chymotrypsin contains 12.7% basic amino acid residues. If CG electrostatically interacts with these proteoglycans in MCF-7 cells, preincubation of CG with anionic molecules, such as sodium dextran sulfate and sodium heparin or cationic molecules, including lactoferrin, would be expected to inhibit the binding. Contrary to our expectations, pretreatment of ^125^I-CG (83.4 nM) with 250 *μ*g/mL sodium dextran, 250 *μ*g/mL sodium heparin, or 20 *μ*M lactoferrin did not inhibit CG binding to MCF-7 cells (data not shown). Our results indicate that the binding of CG is mediated by a receptor-like molecule. 

 The relationship between the concentrations of CG and inhibitory factors such as serpins in a tumor mass is very interesting. It is thought that a large portion of CG is inactivated by serpins in blood. CG is inhibited by serpins via a suicide substrate inhibition mechanism [3, 28]. After cleavage, CG forms a covalent complex with the cleaved serpin peptide, and finally CG is denatured. Indeed, the cell aggregation-inducing activity of CG is inhibited by serpins such as AT or ACT. The human serum concentration of ACT is approximately 0.25 mg/mL (3.7 *μ*M) and increases by almost 5-fold during acute inflammation [46]. Although the amount of serpins present in tumors is not known, approximately 80 *μ*g (3.3 nmol) of CG is reported to be present in 10^8^ human polymorphonuclear neutrophils (PMNs) [47, 48]. Given that 1 nM CG weakly induces MCF-7 cell aggregation (Figure 1), the local concentration of CG may be sufficient to function in inflammation sites and tumor tissues where neutrophils accumulate and secrete proteases in azurophil granules. *In vivo* experiments are currently being designed to address these possibilities. 

 In conclusion, we propose that the molecular mechanism of CG-induced MCF-7 cell aggregation entails 2 steps: the interaction between CG and a cell surface molecule on MCF-7 cells and the proteolytic cleavage that induces cell aggregation. Further studies are underway in our laboratory to further elucidate these interactions.

## Supplementary Material

Covalent complex formation between CG and AT or ACT: CG purified from neutrophils (1.67 pmol) was incubated with AT (90.9 pmol) or ACT (1.5 pmol) in RPMI 1640 medium containing 1% BSA for 10 min at 4°C. In addition, to measure the effect of an inhibitor on complex formation, CG was pretreated with chymostatin (661 pmol). Formation of the covalent complex in the mixture was determined by the presence of a heavier CG band on the western blot membrane.
Supplementary figure 1: Recombinant human CG is enzymatically active. Recombinant CC was prepared from RBL-2H3 cells that transfected with the vector encoding WT or S195G human CG cDNA. A: Enzymatic activity of CG in the cell lysates. The release rate of 4-nitroanilides was measured by adding 20-*μ*L of the cell lysate to N-succinyl Ala-Ala-Pro-Phe *p*-nitroanilide in 0.1 M HEPES-NaOH (pH 7.5) containing 0.5 M NaCl and 10% dimethyl sulfoxide (180 *μ*L) at 25°C. The released *p*-nitroanilide was detected by the absorbance at 405 nm. The results are shown as mean ± SD (n = 3). B: immunoreactivities of recombinant CG and *β*-actin in the lysates assayed in Supplementary fig. 1A. A 10-*μ*L aliquot of the whole cell lysate were analyzed by western blotting using anti-CG and *β*-actin antibody. Lane 1, purified CG from human neutrophils (100ng); lane 2, the lysate of cells transfected with pcDNA3.1 that encodes of WT CG; lane 3, the lysate of cells transfected with pcDNA3.1 that encodes S195G CG; lane 4, the lysate of vector-transfected cells. The ratio of CG and *β*-actin protein contained in the lysates, which were quantified using ImageQuant TL, are indicated under the photographs. C: The enzymatic activity after 80 min was normalized using *β*-actin. The results are shown as mean ± SD (n = 3). When the bars are not shown, they are smaller than the size of the symbols.
Supplementary figure 2: Complex formation of CG and AT or ACT is abrogated by pre-treatment with chymostatin. Purified CG was incubated with AT or ACT for 10 min at 4°C. CG was pretreated with chymostatin to assess its inhibitory effect on complex formation. The formation of a covalent complex of CG and AT (or ACT) in the mixture was analyzed by western blotting using anti-CG antibody. CG, 26 kDa; AT, 50 kDa; ACT, 50 kDa.
Supplementary figure 3: PARs did not participate in MCF-7 cell aggregation induced by cathepsin G. MCF-7 cells were inoculated 1 × 10^4^ cells/well in 96 well plate in RPMI 1640 medium supplemented with 5% FBS. The cells were cultured overnight and then incubated with a peptide of PAR-1 (A), PAR-2 (B) or PAR-4 (C) ligand in RPMI 1640 medium containing 1% BSA overnight. After washing, the residual cells were stained with crystal violet, and the aggregation index was calculated. The results are expressed as mean ± SD (n = 3). When the bars are not shown, they are smaller than the size of the symbols.
Supplementary figure 4: Scatchard plot of 125I-labeled CG binding to the MCF-7 cells.
