# Supplementary material for: Cathepsin G Induces Cell Aggregation of Human Breast Cancer MCF-7 Cells via a 2-Step Mechanism: Catalytic Site-Independent Binding to the Cell Surface and Enzymatic Activity-Dependent Induction of the Cell Aggregation
Source: Mediators Inflamm. 2012 Jul 8;2012:456462. doi: 10.1155/2012/456462 (PMC3418687; doi:10.1155/2012/456462)

Supplementary fig. 1

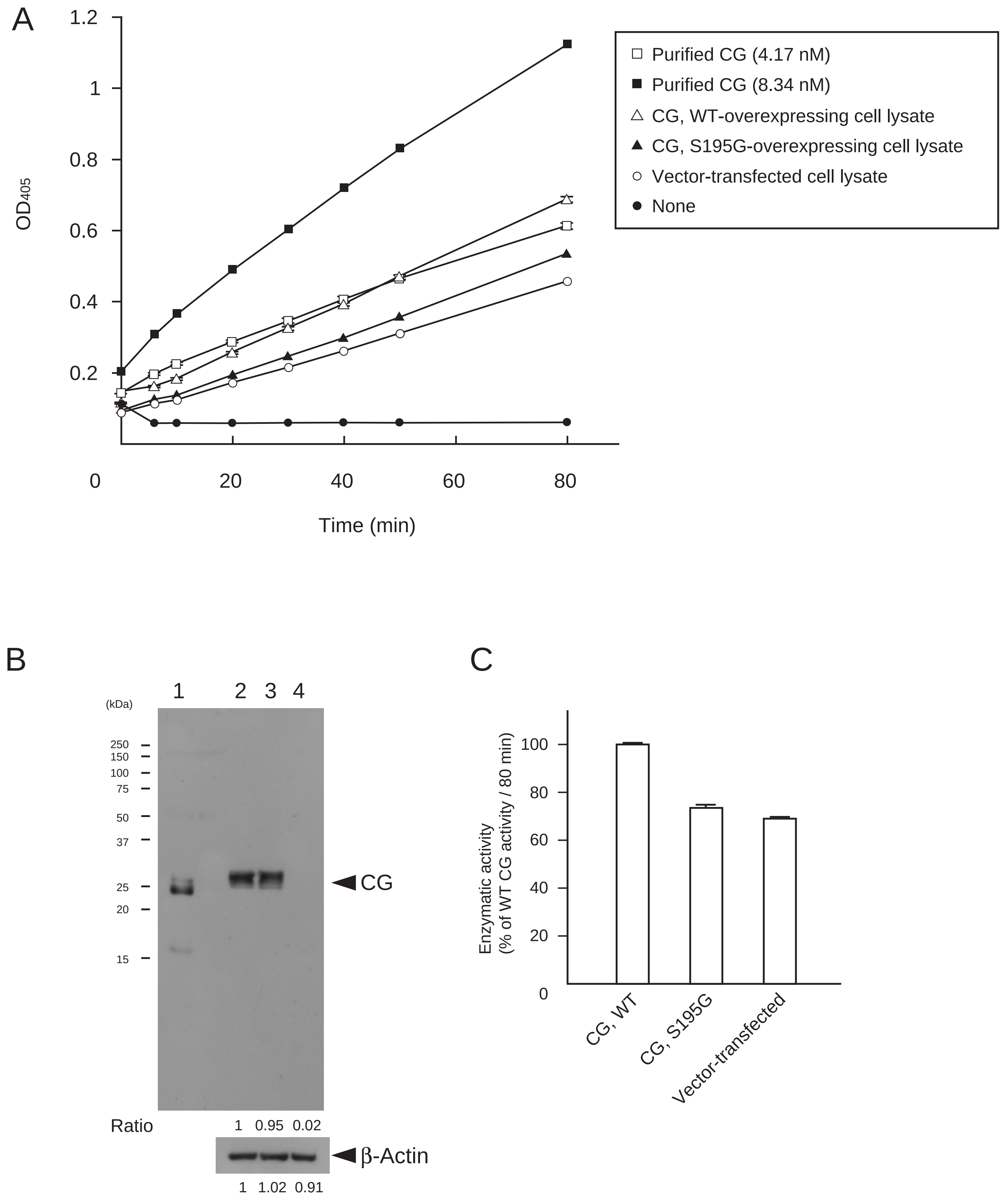

Supplementary fig. 2

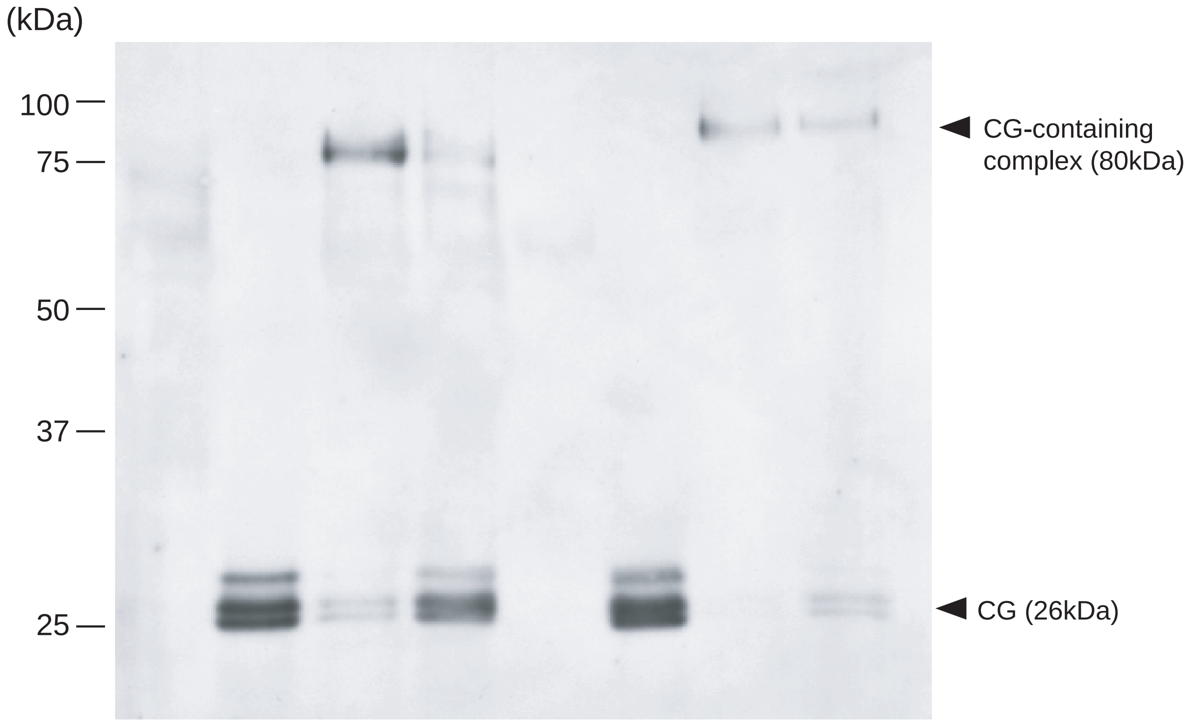

CG (pmol)	-	1.67	1.67	1.67	-	1.67	1.67	1.67
AT (pmol)	90.9	-	90.9	90.9	-	-	-	-
ACT (pmol)	-	-	-	-	1.5	-	1.5	1.5
Chymostatin (pmol)	-	-	-	661	-	-	-	661

Supplementary fig. 3

A PAR-1 agonist

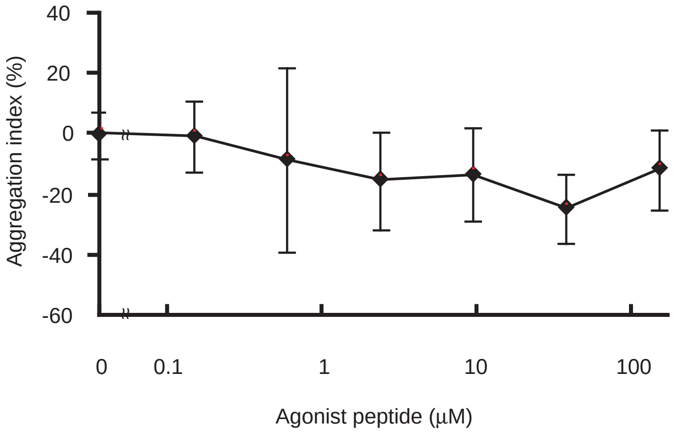

B PAR-2 agonist

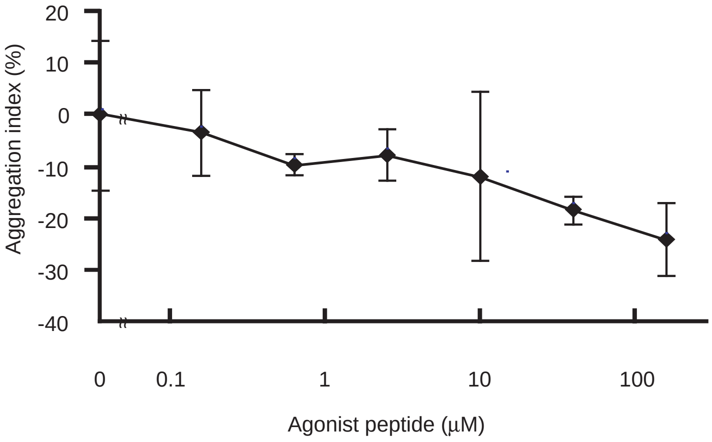

C PAR-4 agonist

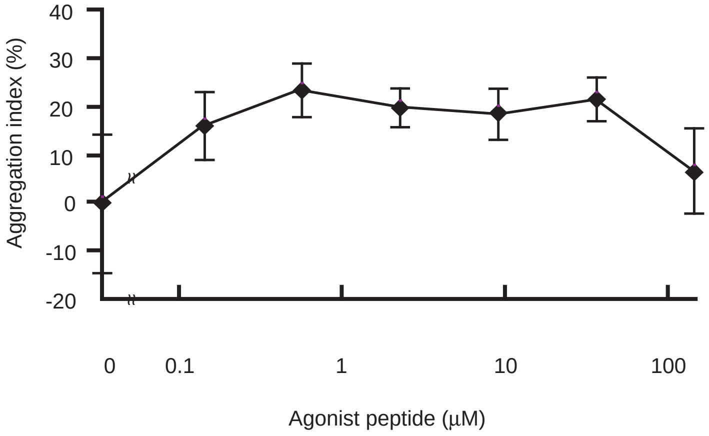

Supplementary fig. 4

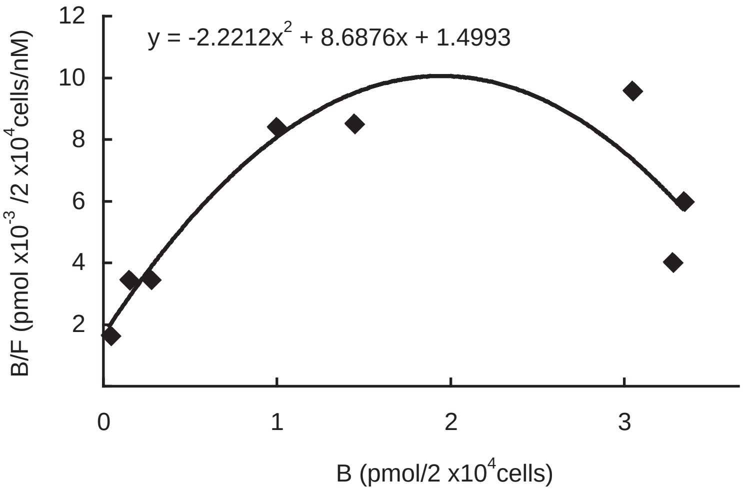

Supplement: Supplementary file 1 — Covalent complex formation between CG and AT or ACT: CG purified from neutrophils (1.67 pmol) was incubated with AT (90.9 pmol) or ACT (1.5 pmol) in RPMI 1640 medium containing 1% BSA for 10 min at 4°C. In addition, to measure the effect of an inhibitor on complex formation, CG was pretreated with chymostatin (661 pmol). Formation of the covalent complex in the mixture was determined by the presence of a heavier CG band on the western blot membrane. Supplementary figure 1: Recombinant human CG is enzymatically active. Recombinant CC was prepared from RBL-2H3 cells that transfected with the vector encoding WT or S195G human CG cDNA. A: Enzymatic activity of CG in the cell lysates. The release rate of 4-nitroanilides was measured by adding 20-μL of the cell lysate to N-succinyl Ala-Ala-Pro-Phe p-nitroanilide in 0.1 M HEPES-NaOH (pH 7.5) containing 0.5 M NaCl and 10% dimethyl sulfoxide (180 μL) at 25°C. The released p-nitroanilide was detected by the absorbance at 405 nm. The results are shown as mean ± SD (n = 3). B: immunoreactivities of recombinant CG and β-actin in the lysates assayed in Supplementary fig. 1A. A 10-μL aliquot of the whole cell lysate were analyzed by western blotting using anti-CG and β-actin antibody. Lane 1, purified CG from human neutrophils (100ng); lane 2, the lysate of cells transfected with pcDNA3.1 that encodes of WT CG; lane 3, the lysate of cells transfected with pcDNA3.1 that encodes S195G CG; lane 4, the lysate of vector-transfected cells. The ratio of CG and β-actin protein contained in the lysates, which were quantified using ImageQuant TL, are indicated under the photographs. C: The enzymatic activity after 80 min was normalized using β-actin. The results are shown as mean ± SD (n = 3). When the bars are not shown, they are smaller than the size of the symbols. Supplementary figure 2: Complex formation of CG and AT or ACT is abrogated by pre-treatment with chymostatin. Purified CG was incubated with AT or ACT for 10 min at 4°C. CG wa [file 456462.f1.pdf]
